# Nutrient Recovery from Algae Using Mild Oxidative Treatment and Ion Exchange

**DOI:** 10.1021/acssuschemeng.4c02658

**Published:** 2024-05-20

**Authors:** Tobias C. Hull, Kameron J. King, Jacob S. Kruger, Earl D. Christensen, Ali Chamas, Philip T. Pienkos, Tao Dong

**Affiliations:** 1 Advanced Energy Systems Graduate Program, Colorado School of Mines, Golden, Colorado 80401, United States; 2 Renewable Resources and Enabling Sciences Center, National Renewable Energy Laboratory, Golden, Colorado 80401, United States; 3 Department of Civil and Environmental Engineering, Old Dominion University, Norfolk, Virginia 23529, United States; 4 Catalytic Carbon Transformation & Scale-Up Center, National Renewable Energy Laboratory, Golden, Colorado 80401, United States; 5 Matereal, Inc. 1007 Savannah Avenue, Pittsburgh, Pennsylvania 15227, United States

**Keywords:** Algae, Biomass, Nutrient recovery, Wet oxidation, Ion exchange, Biofuel

## Abstract

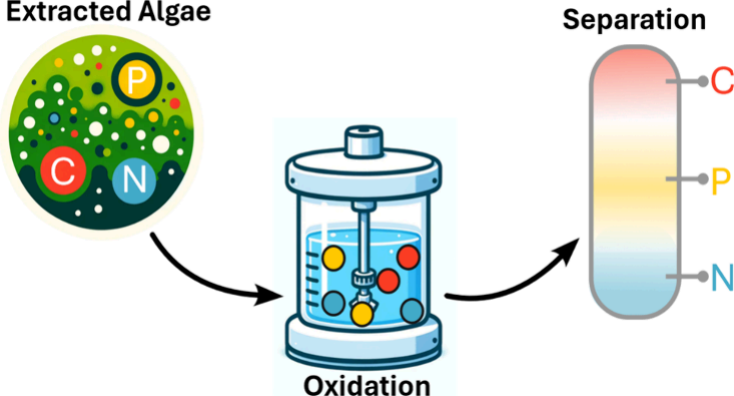

Valorization of algal biomass to fuels and chemicals frequently requires pretreatment to lyse cells and extract lipids, leaving behind an extracted solid residue as an underutilized intermediate. Mild oxidative treatment (MOT) is a promising route to simultaneously convert nitrogen contained in these residues to easily recyclable ammonium and to convert carbon in the same fraction to biofuel precursor carboxylates. We show that for a *Nannochloropsis* algae under certain oxidation conditions, nearly all the nitrogen in the residues can be converted to ammonium and recovered by cation exchange, while up to ∼20% of the carbon can be converted to short chain carboxylates. At the same time, we also show that soluble phosphorus in the form of phosphate can be selectively recovered by anion exchange, leaving a clean aqueous carbon stream for further upgrading.

## Abstract 2

We developed a process to recover nutrients from a waste stream to improve sustainability during algal biorefining.

## Introduction

Algal biomass is a sustainable carbon source capable of advancing decarbonization efforts in the fuel and chemical industries. Algae are known for their capacity to produce large quantities of energy-dense lipids,^1^ the extraction and conversion of which is a central point in many algal biorefinery designs. Extraction residues are rich in nitrogen and phosphorus, making the development of efficient nutrient recovery methods from them necessary for a sustainable algae industry.^2^ To our knowledge, the only published strategies for nutrient recovery from extracted algal biomass have focused on anaerobic digestion (AD)^3−10^ to produce a nutrient-rich aqueous digestate suitable for recycling to cultivation ponds. AD also generates biogas which can offset some energy demand of the algal biorefinery.^11^ However, AD of extracted algae has some limitations, including limited throughput and sensitivity to methods used for the initial pretreatment and lipid extraction processes. Therefore, we were motivated to explore alternative methods for nitrogen and phosphorus nutrient recovery and carbon valorization from extracted algae residues.

Extracted algal residues are produced in fractionation-based approaches to algal conversion, such as the combined algal processing (CAP) pathway.^12,13^ CAP uses a dilute acid pretreatment and solvent extraction to fractionate wet algal biomass into three distinct intermediate phases: an organic solvent containing extracted lipids, an aqueous hydrolysate enriched with soluble carbohydrates and proteins, and a residual solid phase comprised of the extracted algal residues.^14^ Processes to convert extracted lipids into renewable hydrocarbon fuels and non-isocyanate polyurethanes and ferment aqueous hydrolysates into other bioproducts are established, but the valorization of residual extracted solids remains underdeveloped.^13,15,16^ These solids, often viewed as a lower-value substrate due to their high content of non-food-grade protein, contain substantial amounts of nitrogen and phosphorus, primarily bound in proteins and complex polyphosphates.^17^ To extract and recover nitrogen and phosphorus nutrients from these solids, we developed a process using oxidation to convert these nutrients into ionic forms, making them suitable for recovery through ion exchange techniques.

Selective oxidative deamination of amino acids in extracted algae residues has been proposed as a method to enable nutrient recovery, yet remains undemonstrated.^12^ In physiological systems, amino acids can undergo oxidative deamination, producing ammonium and a carboxylate one carbon shorter than the parent amino acid.^18,19^ This mechanism is the primary pathway for amino acids with alkane side chains; additional pathways exist for more complex side chains, and the products may also be subsequently converted to smaller acids and CO_2_. These reports use Fenton’s reagent to generate hydroxyl radicals to initiate oxidation. Similar radicals are believed to initiate noncatalytic wet air oxidation processes in high-temperature water,^20^ and amide bonds are also cleaved under these conditions, promoting protein depolymerization.^21^ Therefore, we hypothesized that partial wet air oxidation under relatively mild conditions, or mild oxidative treatment (MOT), of extracted algae solids could convert proteins to ammonium and a mixture of carboxylates while concurrently hydrolyzing polyphosphates. This process is expected to extract nutrients from algal residues, converting them into ionic forms to simplify their subsequent recovery.

Although techniques to recover ammonium and phosphate from aqueous systems are well-established, not all are suitable for a biorefinery setting. Struvite precipitation, air stripping, and adsorption rank among the leading options for nutrient recovery from wastewater.^22^ However, air stripping requires an alkaline environment to shift equilibrium toward gaseous NH_3_, which may be suboptimal given the acidic nature of solutions generated by acid pretreatment in the CAP process. Precipitation methods are also pH-sensitive, and the presence of interfering carboxylate ions can compromise recovery yields.^23,24^ Given these constraints, ion exchange stands out as a promising nutrient recovery technique. Ion exchange operates through the reversible exchange of charged species between a stationary matrix and a mobile phase, enabling the targeted extraction of ions from the mobile phase onto the matrix.^25^ Subsequently, the ions can be recovered from the matrix and collected in the effluent during a regeneration cycle, providing a straightforward, sustainable, and cost-effective pathway for nutrient recycle.^26^ Therefore, we further hypothesized that the ionic ammonium and phosphate would be amenable to selective recovery from the MOT product liquor, while the mixture of carboxylates would be amenable to valorization as sustainable aviation fuel through ketonization, condensation, and hydrogenation.^11,27^

**Figure 1:**
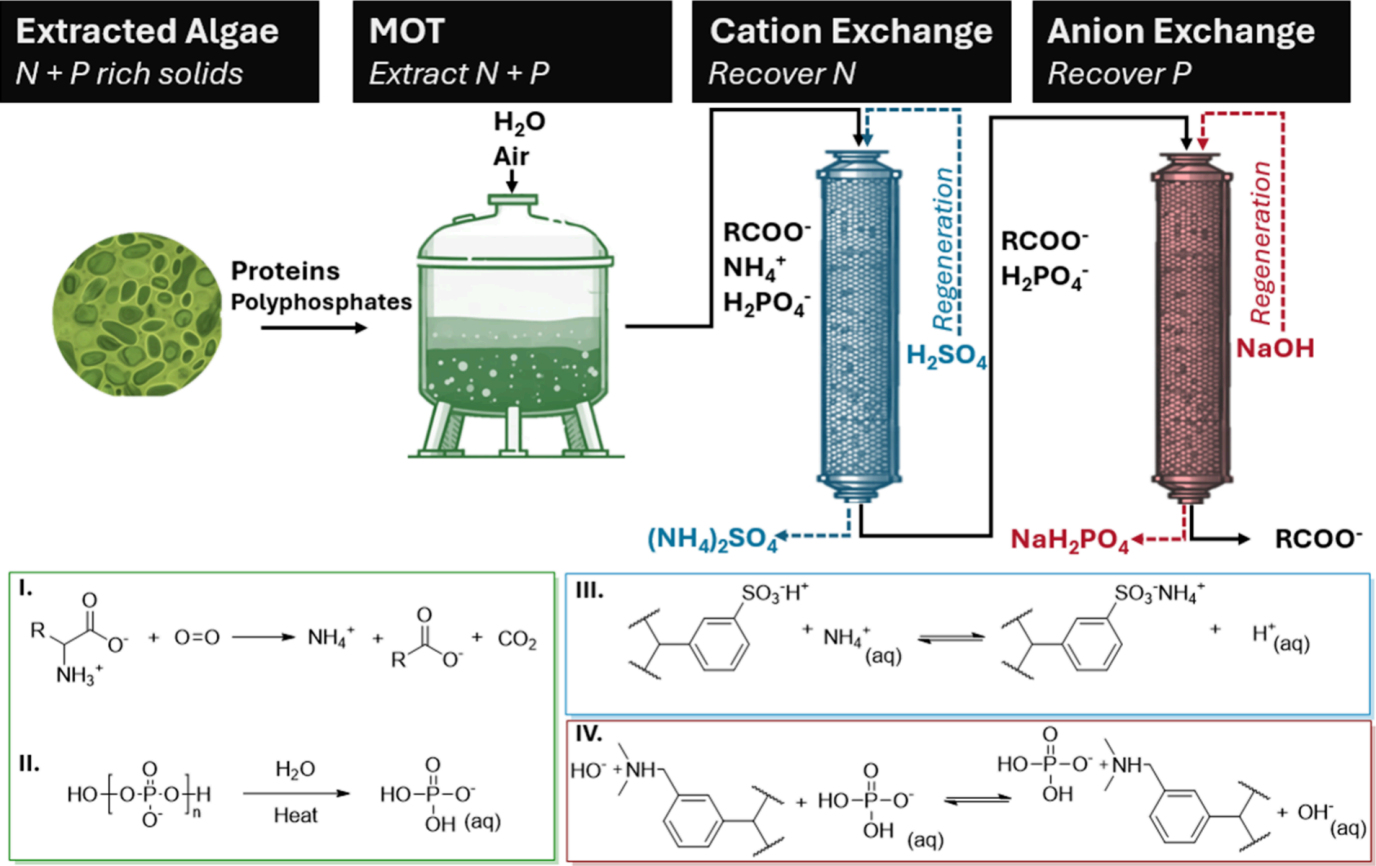
Schematic representation of the conversion of nutrients in extracted algae into recoverable ammonium and phosphate via MOT, followed by their subsequent separation and recovery using ion exchange resins. Key reactions in each stage are highlighted: (I) oxidative deamination of aliphatic amino acids; (II) hydrolysis of polyphosphates; (III) recovery of ammonium on a sulfonic acid functionalized strong acid cation exchange resin; (IV) recovery of phosphate on a tertiary amine functionalized weak base anion exchange resin.

This study investigates the integrated production of ammonium nitrogen, phosphate, and short chain carboxylates through MOT of extracted algae solids, summarized schematically in Figure 1. Nitrogen, expected to be present as ammonium (NH_4_^+^) in the form of ammonium carboxylate salts after MOT, is targeted for recovery by cation exchange. Phosphorus, presumed to be present as phosphate anions (PO_4_^3–^, HPO_4_^2–^, and H_2_PO_4_^–^), is aimed to be selectively recovered by anion exchange. The integration of MOT with ion exchange presents a new approach to nutrient recovery and carbon valorization from extracted algal solids.

## Materials and Methods

### Preparation of Extracted Algae Solids

*Nannochloropsis* sp. was donated by an industrial partner as a dry algal flake and stored at 4 °C prior to processing. The biomass was processed as described previously.^28^ Briefly, the extracted solids were prepared by dilute acid pretreatment of a 20 wt % algal biomass slurry with 1 wt % H_2_SO_4_ (g/g algal biomass) at 175 °C for 15 min in a Zipperclave reactor. The acidified pretreated slurry was separated by centrifugation, and lipids were extracted from the pretreated solids by a mixture of ethanol and hexane. The extraction process used a mass ratio of pretreated solids:hexane:ethanol of 3:3:1 and was conducted at room temperature for 2 h per extraction. Following extraction, the mixture was centrifuged and the organic phase removed. The resulting extracted algae solid solids were sequentially dried under air and in a vacuum oven at 40 °C, ground to <20 mesh in a Wiley mill, and stored in a freezer at −20 °C until use.

### Compositional Analysis

The *Nannochloropsis* extracted solids were analyzed for ash, lipid (as fatty acid methyl esters), and carbohydrates based on National Renewable Energy Laboratory standard Laboratory Analysis Procedures (LAPs).^29−32^ Briefly, ash content was determined by combusting the substrate,^30^ lipids were determined using an *in situ* transesterification procedure,^31^ and total carbohydrate content was determined through a two-stage hydrolysis procedure, followed by HPLC analysis.^32^ The total carbon, hydrogen, and nitrogen content of the samples was determined by combustion using a LECO TruSpec CHN module. Total phosphorus was determined by ICP after acid digestion (US EPA 200.7). The amino acid profile was determined after acid hydrolysis by ion exchange chromatography with postcolumn ninhydrin derivatization (AOAC 994.12). All values are reported as weight percent of the dry sample.

### MOT Reactions

MOT reactions were carried out on a series 5000 multiple reactor system from Parr Instruments. This setup included six 75 mL batch reactors in parallel with temperature, pressure, and magnetic stirring controls for each vessel. In a typical reaction, extracted algal solids were mixed with 25 mL of deionized water to achieve the desired solids loading (20–200 g/L). The slurry was loaded into a 316 SS batch reactor, along with a stir bar. The reactors were purged with ultrahigh purity (UHP) helium three times and then leak tested. Upon passing a leak test, reactors were depressurized to 1 bar of He. Reactors were then heated to the desired temperature (175–250 °C) at which point the desired partial pressure of oxygen (1–8 bar) was introduced to the system by adding UHP zero air, marking the 0 min time point for these reactions. Adding the oxidant at temperature was done to avoid oxidation effects during heat up, which typically took around 30 min. The reaction proceeded for the desired time (5–60 min) after the addition of oxygen, at which point the reactors were quenched in an ice bath. Once cool, reactors were depressurized, and the product solution was vacuum filtered to separate residual oxidation solids from the MOT liquor. The postoxidation solids were further dried in a vacuum oven at 40 °C prior to analysis.

### Ion Exchange Studies

Amberlite IRC-120 H (hydrogen form) was purchased from Sigma-Aldrich, and Amberlite IRA-67 was purchased from GFS Chemicals. Both resins were washed with UHP water until the pH of the effluent was neutral in order to remove surface impurities. After washing, resins were dried in a vacuum oven and stored in a desiccator until use. Ion exchange reactions were carried out in batch mode at room temperature under gentle stirring. After each ion exchange step, the ion exchange material was recovered from the solution by centrifugation, and an aliquot of the ion exchanged solution was reserved for analysis.

### Product Analysis

Total nitrogen was quantified by combustion analysis or by N chemiluminescence (ASTM D4629), depending on the level. Total phosphorus was determined by ICP after acid digestion (US EPA 200.7). Aqueous organic products, including amino acids, carboxylates, and ammonium, were analyzed by a propyl chloroformate (PCF) derivatization, followed by GC–MS analysis. The derivatization procedure was a modified version of Villas-Bôas et al.’s methyl chloroformate (MCF) derivatization wherein PCF and 1-propanol were substituted for MCF and methanol, and derivatized compounds were extracted into diethyl ether rather than chloroform.^33^ These modifications facilitated separation of derivatized formic and acetic acid from each other and from the solvent peak. Kaspar et al. had previously demonstrated the efficacy of PCF as a derivatizing agent for the analysis of free amino acids.^34^ An Agilent 6890 GC coupled with a 5973 mass selective detector (MSD) equipped with a 30 m, 0.25 mm i.d. RTX-50 capillary column was used for the GC–MS analyses. The initial oven temperature was set to 50 °C and then increased to 140 °C at a rate of 7 °C/min. The ramp rate was then increased to 12 °C/min until the temperature reached 300 °C, where it was held for 5 min. The flow through the column was 1 mL of He/min. The injection volume was 1 μL, and the split ratio was 1:20. An internal standard of D7-butyric acid and D3-alanine was added to the analyte prior to derivatization and used for quantification of the derivatized carboxylate and amino acid peaks, respectively.

## Results and Discussion

### Compositional Analysis of Extracted Algae

After dilute acid pretreatment and lipid extraction, the remaining extracted algae solids were collected, lyophilized, and analyzed. The upstream processes effectively extracted the majority of carbohydrates and lipids from the biomass, leaving behind a solid residue consisting mostly of protein and ash, as reported in Table 1. The low mass closure of the compositional analysis indicates that some of the components were likely degraded during pretreatment, producing derivatives that were not recognized. A detailed breakdown of the lipid, protein, and carbohydrate profiles is provided in the Supporting Information Tables S1–S3. Notably, by summing quantifiable amino acids, it is apparent that 28% of the carbon and 55% of the nitrogen in the extracted solids are present as protein. Due to the low mass closure from the compositional analysis, we conservatively report MOT product yields based on molar amounts of carbon, nitrogen, or phosphorus in the extracted algae solids.

**Table 1:** Composition and Elemental Profile of the Extracted *Nannochloropsis* Solids Used for the MOT Reactions

compositional analysis (wt %)	
lipids	3.6
carbohydrates	1.5
proteins^a^	27.7
ash	35.5

elemental analysis (wt %)	
carbon	37.3
hydrogen	5.2
nitrogen	5.8
phosphorus	0.7

a Calculated as 4.78 × N.

### MOT of Extracted Algae

**Figure 2:**
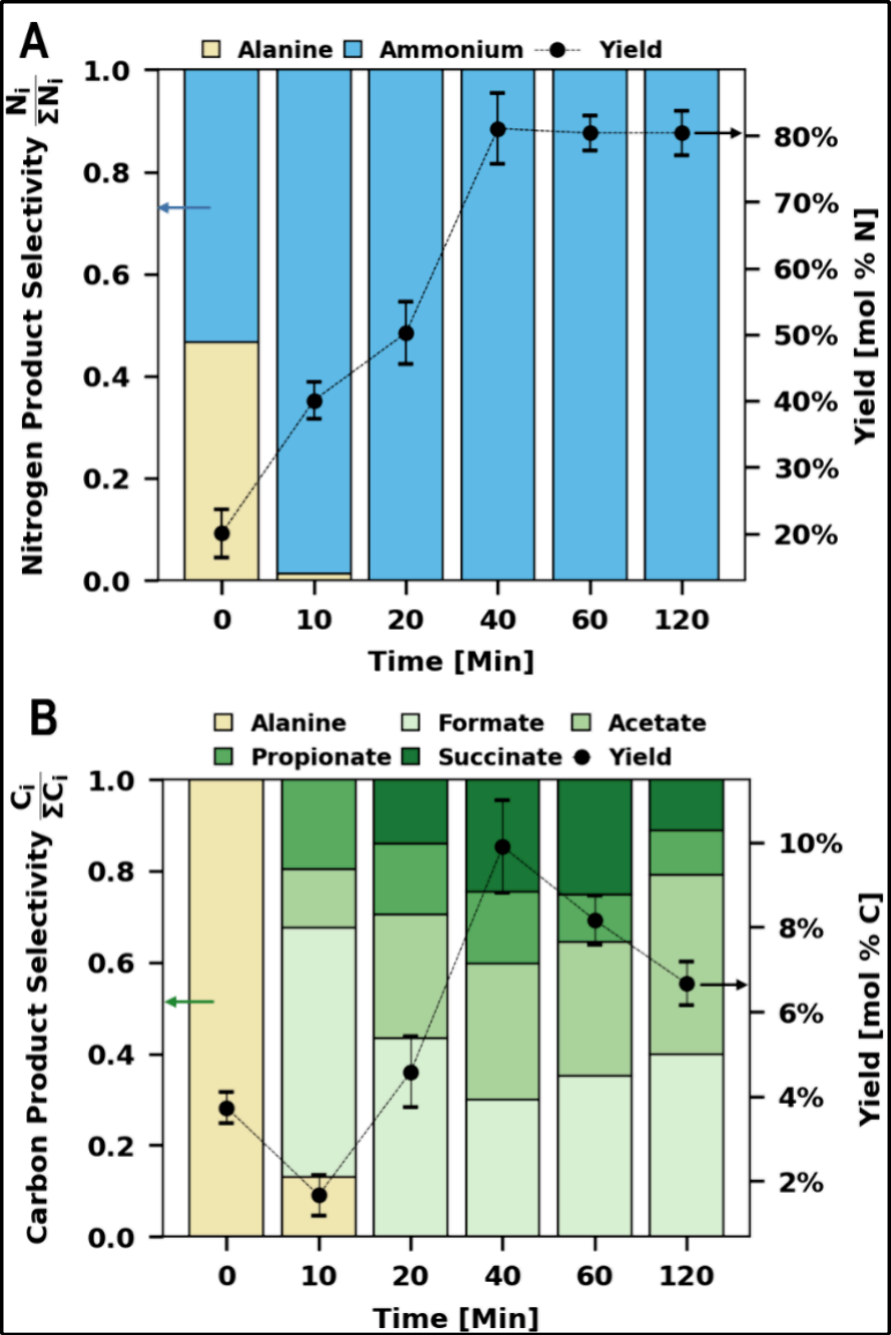
Product distribution during MOT of extracted algae solids, illustrating nitrogenous (A) and carbonaceous (B) products in the MOT liquor over time. The bar chart on the left *y*-axis shows product selectivity. The line chart on the right *y*-axis shows combined yields as the ratio of moles of nitrogen or carbon in products to those in extracted solids. Error bars show the standard deviation of combined molar yields. Reaction conditions: 0.5 g of dry solids, 25 mL of H_2_O, 200 °C, 2 bar O_2_ partial pressure added upon reaching reaction temperature, 40 min reaction time. Corresponding numeric data and product concentrations are included in Supporting Information Tables S4 and S5.

The product profile in the MOT liquor over the course of a typical MOT of extracted algal solids is shown in Figure 2. The 0 min time point reflects conditions as the reactor reaches the desired temperature but prior to the addition of air. Both ammonium and alanine are detected at this point, likely due to algal protein hydrolysis in subcritical water during heat-up.^35^ Upon introducing oxygen, carboxylate and additional ammonium production begins, coupled with a reduction in alanine concentrations, leaving no amino acids after 10 min. Subsequently, ammonium remains as the only nitrogenous product, while detected carboxylate products are distributed across formate, acetate, propionate, and succinate. Product concentrations increase over time, reaching molar yields of 80 mol % nitrogen for ammonium and 10 mol % carbon for carboxylates by the 40 min mark. Ammonium concentrations are stable beyond this point, but carboxylates exhibit signs of degradation, likely lost as CO_2_.^36,37^

The origin of MOT products, whether via the oxidation of hydrolyzed amino acids or from other biomass components, remains uncertain. Ammonium yields exceed individual protein and non-protein nitrogen content of the extracted solids, suggesting origins from both biomass fractions. In contrast, carboxylate yields are lower. The oxidation of both protein and non-protein fractions can produce acids, yet inherently results in some carbon lost as CO_2_. A first-order approximation based on quantified amino acids predicts a theoretical yield of 20 mol % carbon to carboxylates from the protein fraction. Predicting carbon yields from non-protein fractions is more complex. Kinetic models for wet oxidation processes sometimes categorize carbon into “fast” reacting fractions, which quickly convert to CO_2_, and “slow” reacting ones that form refractory acids like acetic acid.^38^ Matching these fractions with specific, measurable compositions, however, remains challenging.

**Figure 3:**
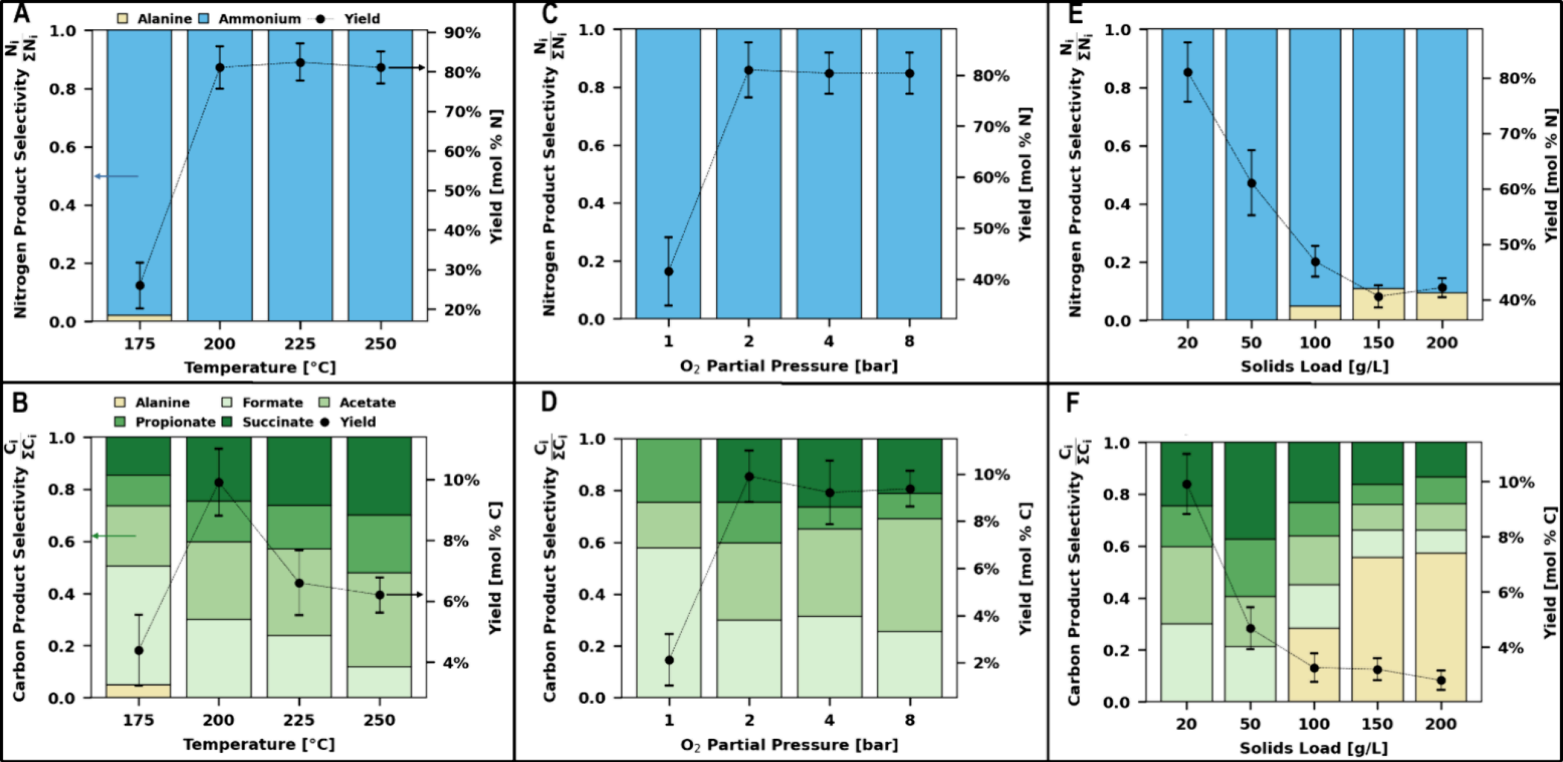
Product distribution during MOT of extracted algae solids illustrating nitrogenous (A, C, E) and carbonaceous (B, D, F) products in the MOT liquor at various temperatures (A, B), oxygen partial pressures (C, D), and solid loadings (E, F). The bar charts on the left *y*-axes show product selectivity. The line charts on the right *y*-axes shows combined yields as the ratio of moles of nitrogen or carbon in products to those in extracted solids. Error bars show the standard deviation of combined molar yields. Reaction conditions unless otherwise noted: 0.5 g of dry solids, 25 mL of H_2_O, 200 °C, 2 bar O_2_ partial pressure added upon reaching reaction temperature, 40 min reaction time. Corresponding numeric data and product concentrations are included in Supporting Information Tables S6–S11.

We further surveyed several MOT reaction conditions by varying temperatures from 175 to 250 °C, oxygen partial pressures from 1 to 8 bar, and solid loading from 20 to 200 g/L. These selected conditions represent a milder regime for the oxidation process relative to conventional wet oxidation processes, ideally leading to the stabilization of intermediate acids rather than their deconstruction. A selection of results, taken after 40 min reaction time, is displayed in Figure 3. Temperature effects on ammonium yields (Figure 3A,B) were negligible above 200 °C, with yields reaching around 80 mol % nitrogen in all cases. Carboxylate yields were similarly insensitive to temperature changes with a maximum yield of about 10 mol % carbon, though at higher temperatures carboxylate concentrations peaked before 40 min and degraded over time, particularly with regards to the loss of formic acid.

Neither ammonium nor carboxylate yields were majorly affected by increased oxygen partial pressures beyond a minimal threshold of 2 bar O_2_ (Figure 3C,D). Kinetic models for the wet oxidation of carboxylates typically report ∼0.5 reaction order with respect to the dissolved oxygen concentration,^36^ and transfer of oxygen from the gaseous to the aqueous phase is not considered a rate limiting step.^38^ Therefore, we suspect that the process may be limited by the hydrolysis of the extracted solids, given the insensitivity to changes in oxygen pressures. To further investigate this, MOT was carried out at increasing solids loading up to 200 g/L (Figure 3E,F). It was found that both nitrogen and carbon product yields decreased with increasing solids loading down to just 2 mol % to carbon products and 40 mol % to nitrogen products at a 200 g/L extracted solids loading, reinforcing the idea that mass transfer of the solid to the aqueous phase was the limiting step in MOT of extracted algae solids.

To address these limitations, 1 wt % H_2_SO_4_ was added to the extracted algal slurry prior to MOT in order to improve solubilization of the substrate. Yields to aqueous phase products increased with the addition of acid, with the yields to ammonium reaching 94 mol % nitrogen and yields to carboxylates nearly doubling to 19 mol % carbon, with a notable increase in the amount of acetate produced. Table 2 provides a complete breakdown of the product spectrum with and without the addition of acid. Further ICP analysis of the acid-treated MOT liquor showed that 77% of total phosphorus contained in the original solids was extracted into the aqueous phase under these conditions.

**Table 2:** Yields to Aqueous MOT Products with and without the Addition of H_2_SO_4_ To Promote the Solubilization of the Substrate^a^

compound	no acid	1 wt % H_2_SO_4_
Carbon Yield (mol % C)		
formate	3.0	4.2
acetate	2.9	8.8
propionate	1.6	3.3
succinate	2.4	2.7
Nitrogen Yield (mol % N)		
ammonium	81.0	94.5

a Reaction conditions unless noted: 0.5 g of dry solids, 25 mL of H_2_O, 200 °C, 2 bar O_2_ partial pressure added upon reaching reaction temperature, 40 min reaction time.

**Figure 4:**
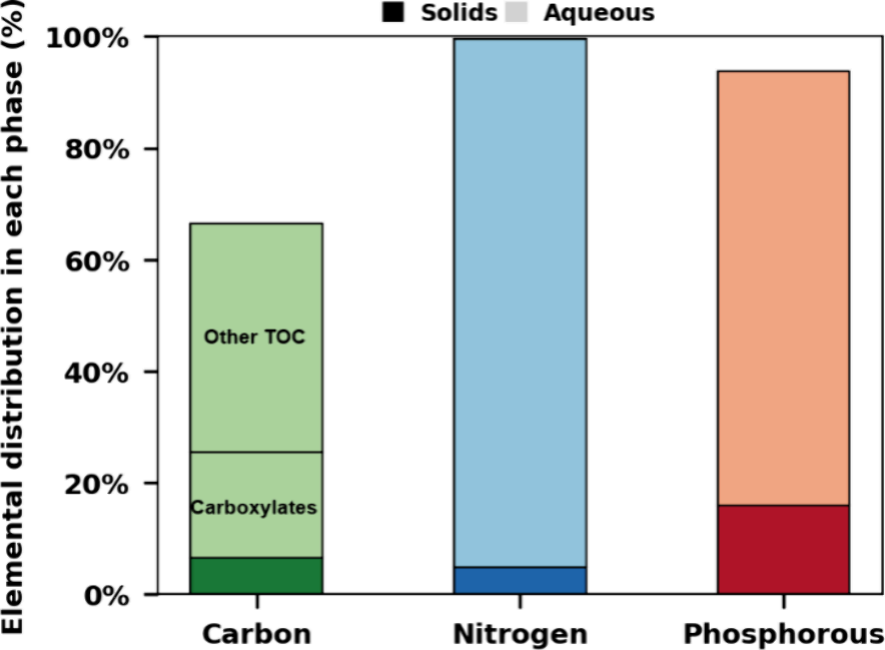
Distribution of carbon, nitrogen, and phosphorus in solid and aqueous phases after MOT of extracted algae solids. The carbon not accounted for in the mass balance is assumed to be lost as CO_2_. Reaction conditions: 0.5 g of dry solids, 25 mL of H_2_O, 1 wt % H_2_SO_4_, 200 °C, 2 bar O_2_ partial pressure added upon reaching reaction temperature, 40 min reaction time.

Given their efficacy, these conditions were chosen to demonstrate the integrated nutrient recovery process, prompting further analysis to ensure closure of carbon, nitrogen, and phosphorus mass balances as reported in Figure 4. Remarkably, TOC analysis revealed that the MOT liquors contained 60% of the total of the carbon in the algal solids, even though only 19% was identifiable as carboxylates in the aqueous phase. The nature of the remaining 41% of carbon in the aqueous phase is yet to be determined, as it was not identifiable as protein, carbohydrates, or lipids in compositional analysis. The postoxidation solids retained 6% of the initial carbon which similarly was not identifiable by compositional analysis. It is assumed that the unaccounted 34% of carbon was lost as CO_2_. The nitrogen balance was near quantitative, with 94% of nitrogen detected in the aqueous MOT product as ammonium and 5% of nitrogen remaining in the postoxidation solids. Phosphorus was distributed between the aqueous (77%) and solids phases (16%) with the remaining 7% of phosphorus unaccounted for in the mass balance.

### Nutrient Recovery by Ion Exchange

Nutrient recovery from the aqueous phase generated by MOT of extracted algae solids was done in two steps: cation exchange for ammonium nitrogen recovery and anion exchange for phosphate recovery. We opted to use Amberlite IRC-120H, a strong acid cation exchange resin, for ammonium recovery, and Amberlite IRA-67, a weak base anion exchange resin, for phosphate recovery. Relevant characteristics of each ion exchange material are reported in Table 3. These resins were selected based on their documented performance in recovering ammonium and phosphate.^39,40^ Moreover, their optimal performance around a pH of 5.5, the native pH of the MOT solutions, allows for their direct use without requiring any additional conditioning of the MOT solutions.

**Table 3:** Characteristics of Ion Exchange Materials Used for the Recovery of Nutrients from MOT Liquors

name	resin type	functional group	exchange capacity (mg/g) [compound]
Amberlite IRC-120H	cation exchange	sulfonic acid	31.5 [ammonium]^39^
Amberlite IRA-67	anion exchange	tertiary amine	126 [phosphate]^40^

We initially benchmarked the performance of these resins using pure component solutions. Both resins measured adsorption capacities for ammonium and phosphorus matched reported values within error. To simulate conditions closer to those in a column setup, where the solution contacts an excess of resin, a load of 100 mg/mL was used in the batch ion exchange studies. This load is expected to remove both ammonium and phosphate while also offering insight into removal of nontarget compounds like carboxylates.

**Figure 5:**
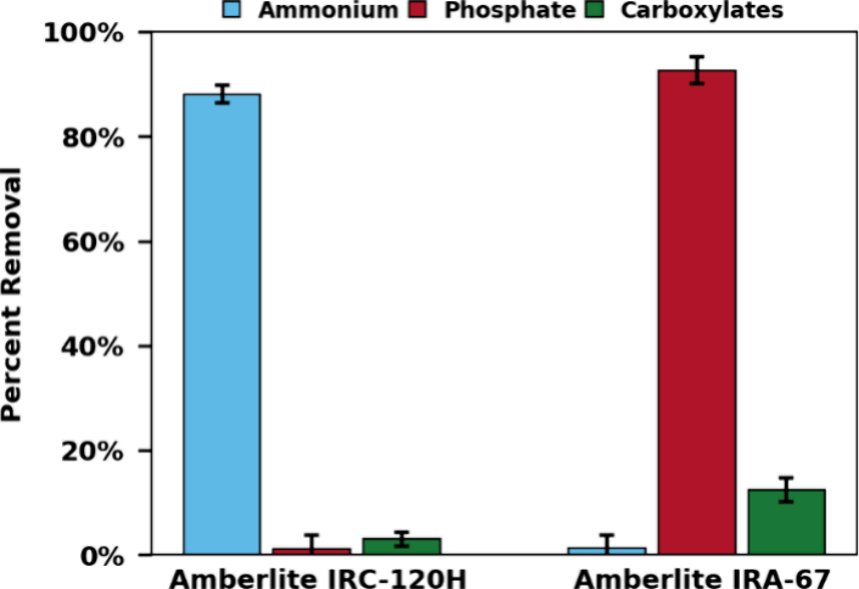
Removal of nutrients and organic acids from MOT liquors by ion exchange resins. Ion exchange conditions: batch process, 100 mg/mL resin load, 60 min contact time, and 100 rpm stir rate. MOT conditions: 0.5 g of dry solids, 25 mL of H_2_O, 1 wt % H_2_SO_4_, 200 °C, 2 bar O_2_ partial pressure added upon reaching reaction temperature, 40 min reaction time.

Ion exchange experiments with authentic MOT liquors, depicted in Figure 5, indicated diminished recovery efficiency by the cation exchange resin. The cation exchange process removed 88% of ammonium. The decrease in performance may stem from competitive adsorption by other cations present in the MOT liquor. Algae cultivated in saltwater retain cations that make up a portion of the ash content. These cations could hinder adsorption due to their affinity for the cation exchange resin.^41^ Notably, the cation exchange resin showed no significant impact on the concentration of carboxylates, enabling the selective recovery of ammonium in the presence of carboxylates.

Anion exchange recovered 92% of phosphorus. It is possible that not all phosphorus present in the MOT liquors is in the form of phosphate and may exist in some speciation that would not interact with the ion-exchange resin. Additionally, some carboxylates were removed. Although anion exchange resins can be used for carboxylic acid recovery, the mechanism primarily involves hydrogen bonding of the protonated acid with the resin, rather than ion exchange of the carboxylate.^42^ Given the MOT solution pH, while the anionic carboxylates are the dominant speciation, some protonated acids are still present. A more selective phosphorus removal may be possible in more alkaline conditions.

### Integrated Process Summary

**Figure 6:**
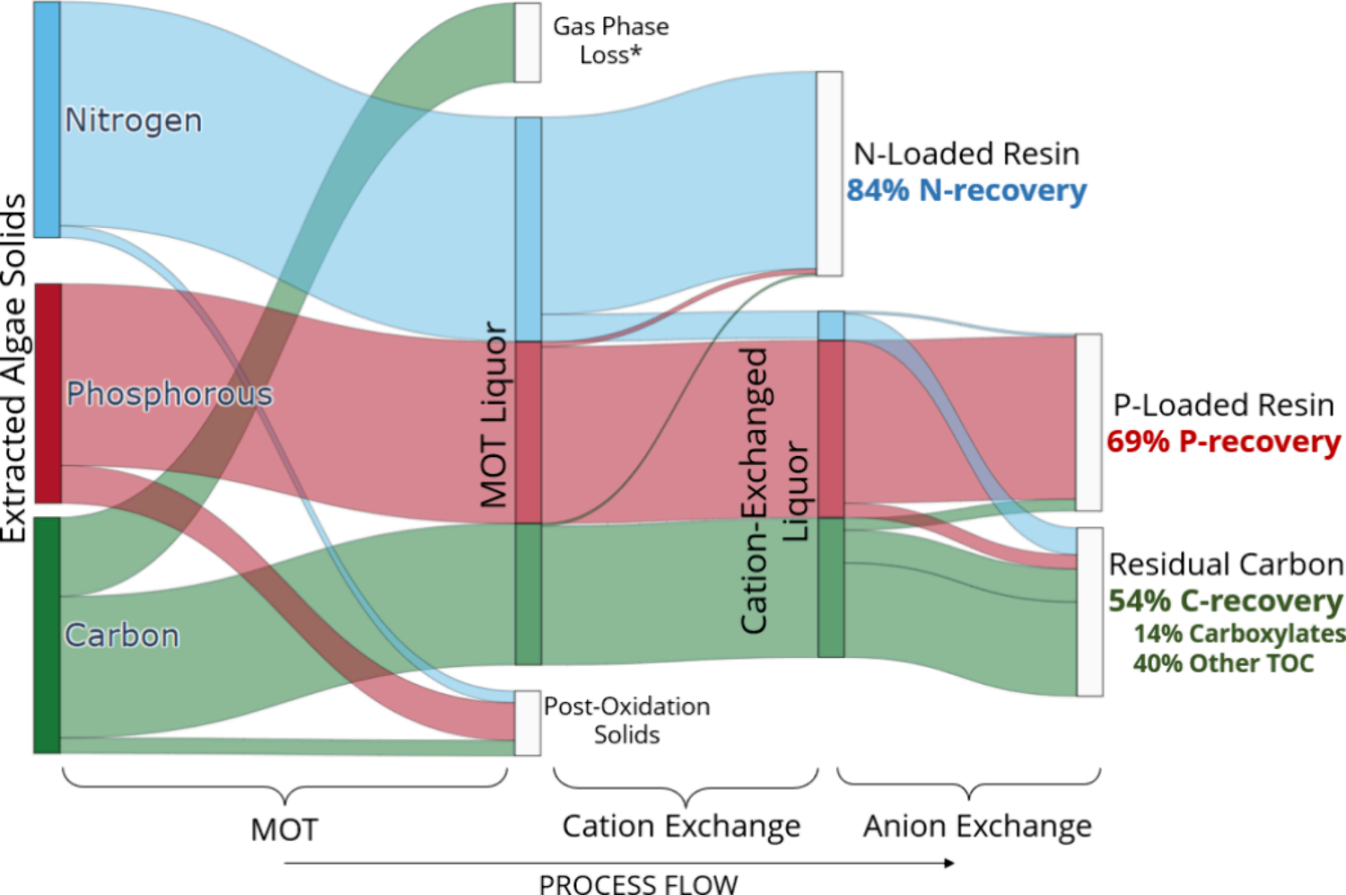
Sankey diagram showing elemental flows during the integrated MOT-ion exchange nutrient recovery process. Flows are expressed as the percentage of each element in the extracted algae solids distributed among product phases after sequential MOT, cation exchange, and anion exchange operations. MOT conditions: 0.5 g of dry solids, 25 mL of H_2_O, 1 wt % H_2_SO_4_, 200 °C, 2 bar O_2_ partial pressure added upon reaching reaction temperature, 40 min reaction time. Ion exchange conditions: batch process, 100 mg/mL resin load, 60 min contact time, 100 rpm stir rate. *Assumed by closing the carbon mass balance.

Building on the MOT and ion exchange experiments, an integrated process to recover nutrients from extracted algae solids was demonstrated. MOT was conducted at a 20 g/L loading of extracted algae solids at 200 °C for 40 min under 2 bar partial O_2_ pressure, with addition of 1 wt % H_2_SO_4_ added to promote solubilization of the substrate. The resulting MOT liquor is previously characterized in Table 2. After MOT, this product solution was filtered and ion exchanged to recover nutrients. Ion exchange involved two sequential cation and anion exchange steps to recover nitrogen as ammonium and phosphorus as phosphate. The process flow and performance summary are illustrated in Figure 6.

The resins performed similarly in the integrated process as in the standalone tests, as reported in Figure 4. In summary, the integrated process was able to recover 84% of the nitrogen in the extracted algae as ammonium and 69% of the phosphorus. At the same time, 54% of the carbon in the extracted solids was retained in the ion-exchanged MOT liquors, 14% of which was identified as carboxylates. Nutrient recovery on synthetic resins implies the need to regenerate the resin for recovery of nutrients and reuse of the resin. The regeneration and reusability of ion exchange resins have been demonstrated for many similar resins in numerous prior publications.^43−45^ For instance, regeneration of the cation exchange resin typically employs aqueous H_2_SO_4_ solution, eluting aqueous (NH_4_)_2_SO_4_. Preliminary experiments with the present resins showed the expected behavior and suggested that, though the process was not optimized in the present experiments, existing commercial technology should suffice for resin regeneration.

There are several options for further valorizing the residual carbon stream, which will be explored in future work. While carboxylates are not currently produced in sufficient quantities to justify a ketonization upgrading approach, future yield enhancements might make this feasible. Alternatively, aqueous carbon may be a useful substrate for fermentation processes, especially ones in which a high carbon-to-nitrogen ratio is desirable, such as those using oleaginous yeasts. While the MOT and ion exchange process was applied to extracted algae solids in this study, it may also be useful to valorize and recover nutrients from other proteinaceous waste streams.

## Conclusions

Nutrient recovery in algae biorefining is necessary to support a sustainable algae industry at the scales required to promote decarbonization efforts. We demonstrated a proof-of-concept process for the recovery of nitrogen and phosphorus from extracted algae residues generated in fractionation-based algal biorefinery designs. The process used partial wet air oxidation under mild conditions (termed mild oxidative treatment or MOT) to convert nutrients bound in algae residues to an easily recoverable aqueous form. Sequential ion exchange over commercially available cation and anion exchange ion exchange resins effectively recovered ammonium and phosphate nutrients from MOT liquors. After ion exchange, MOT liquors retain soluble organic carbon products, including carboxylates, which are promising substrates for further valorization. This work establishes a baseline for a new process for nutrient recovery and carbon valorization from the extracted algae residues.

## Supporting Information Available

Detailed compositional analysis of carbohydrate, amino acid, and lipid profile of extracted algal solids; numeric data for main text figures (PDF)

## References

[ref1] Hu Q.; Sommerfeld M.; Jarvis E.; Ghirardi M.; Posewitz M.; Seibert M.; Darzins A. Microalgal Triacylglycerols as Feedstocks for Biofuel Production: Perspectives and Advances. Plant J. 2008, 54 (4), 621–639. 10.1111/j.1365-313X.2008.03492.x. 18476868

[ref2] Chisti Y. Constraints to Commercialization of Algal Fuels. J. Biotechnol. 2013, 167 (3), 201–214. 10.1016/j.jbiotec.2013.07.020. 23886651

[ref3] Zhao B.; Ma J.; Zhao Q.; Laurens L.; Jarvis E.; Chen S.; Frear C. Efficient Anaerobic Digestion of Whole Microalgae and Lipid-Extracted Microalgae Residues for Methane Energy Production. Bioresour. Technol. 2014, 161, 423–430. 10.1016/j.biortech.2014.03.079. 24736123

[ref4] Bohutskyi P.; Ketter B.; Chow S.; Adams K. J.; Betenbaugh M. J.; Allnutt F. C. T.; Bouwer E. J. Anaerobic Digestion of Lipid-Extracted Auxenochlorella Protothecoides Biomass for Methane Generation and Nutrient Recovery. Bioresour. Technol. 2015, 183, 229–239. 10.1016/j.biortech.2015.02.012. 25746299

[ref5] Bohutskyi P.; Chow S.; Ketter B.; Betenbaugh M. J.; Bouwer E. J. Prospects for Methane Production and Nutrient Recycling from Lipid Extracted Residues and Whole Nannochloropsis Salina Using Anaerobic Digestion. Appl. Energy 2015, 154, 718–731. 10.1016/j.apenergy.2015.05.069.

[ref6] Caporgno M. P.; Clavero E.; Torras C.; Salvadó J.; Lepine O.; Pruvost J.; Legrand J.; Giralt J.; Bengoa C. Energy and Nutrients Recovery from Lipid-Extracted Nannochloropsis via Anaerobic Digestion and Hydrothermal Liquefaction. ACS Sustain. Chem. Eng. 2016, 4 (6), 3133–3139. 10.1021/acssuschemeng.6b00151.

[ref7] Ayala-Parra P.; Liu Y.; Field J. A.; Sierra-Alvarez R. Nutrient Recovery and Biogas Generation from the Anaerobic Digestion of Waste Biomass from Algal Biofuel Production. Renew. Energy 2017, 108, 410–416. 10.1016/j.renene.2017.02.085.

[ref8] Zhang B.; Ogden K. Recycled Wastewater from Anaerobic Digestion of Lipid Extracted Algae as a Source of Nutrients. Fuel 2017, 210, 705–712. 10.1016/j.fuel.2017.09.026.

[ref9] Zhang B.; Ogden K. Nitrogen Balances and Impacts on the Algae Cultivation-Extraction-Digestion-Cultivation Process. Algal Res. 2019, 39, 101434 10.1016/j.algal.2019.101434.

[ref10] Lage S.; Willfors A.; Hörnberg A.; Gentili F. G. Impact of Organic Solvents on Lipid-Extracted Microalgae Residues and Wastewater Sludge Co-Digestion. Bioresour. Technol. Rep. 2021, 16, 100850 10.1016/j.biteb.2021.100850.

[ref11] Wiatrowski M.; Davis R. Algal Biomass Conversion to Fuels via Combined Algae Processing (CAP): 2022 State of Technology and Future Research; NREL, 2023

[ref12] Quiroz-Arita C.; Shinde S.; Kim S.; Monroe E.; George A.; Quinn J.; Nagle N. J.; Knoshaug E. P.; Kruger J. S.; Dong T.; Pienkos P. T.; Laurens L. M. L.; Davis R. W. Bioproducts from High-Protein Algal Biomass: An Economic and Environmental Sustainability Review and Risk Analysis. Sustain. Energy Fuels 2022, 6 (10), 2398–2422. 10.1039/D1SE01230D.

[ref13] Kruger J. S.; Wiatrowski M.; Davis R. E.; Dong T.; Knoshaug E. P.; Nagle N. J.; Laurens L. M. L.; Pienkos P. T. Enabling Production of Algal Biofuels by Techno-Economic Optimization of Co-Product Suites. Front. Chem. Eng. 2022, 3, 803513 10.3389/fceng.2021.803513.

[ref14] Laurens L. M. L.; Nagle N.; Davis R.; Sweeney N.; Van Wychen S.; Lowell A.; Pienkos P. T. Acid-Catalyzed Algal Biomass Pretreatment for Integrated Lipid and Carbohydrate-Based Biofuels Production. Green Chem. 2015, 17 (2), 1145–1158. 10.1039/C4GC01612B.

[ref15] Dong T.; Dheressa E.; Wiatrowski M.; Pereira A. P.; Zeller A.; Laurens L. M. L.; Pienkos P. T. Assessment of Plant and Microalgal Oil-Derived Nonisocyanate Polyurethane Products for Potential Commercialization. ACS Sustain. Chem. Eng. 2021, 9 (38), 12858–12869. 10.1021/acssuschemeng.1c03653.

[ref16] Kruger J. S.; Christensen E. D.; Dong T.; Van Wychen S.; Fioroni G. M.; Pienkos P. T.; McCormick R. L. Bleaching and Hydroprocessing of Algal Biomass-Derived Lipids to Produce Renewable Diesel Fuel. Energy Fuels 2017, 31 (10), 10946–10953. 10.1021/acs.energyfuels.7b01867.

[ref17] Klein B. C.; Davis R. E.; Laurens L. M. L. Quantifying the Intrinsic Value of Algal Biomass Based on a Multi-Product Biorefining Strategy. Algal Res. 2023, 72, 103094 10.1016/j.algal.2023.103094.

[ref18] Stadtman E. R.; Berlett B. S. Fenton Chemistry. Amino Acid Oxidation. J. Biol. Chem. 1991, 266 (26), 17201–17211. 10.1016/S0021-9258(19)47359-6. 1894614

[ref19] Stadtman E. R. Oxidation of Free Amino Acids and Amino Acid Residues in Proteins by Radiolysis and by Metal-Catalyzed Reactions. Annu. Rev. Biochem. 1993, 62 (1), 797–821. 10.1146/annurev.bi.62.070193.004053. 8352601

[ref20] Bhargava S. K.; Tardio J.; Prasad J.; Föger K.; Akolekar D. B.; Grocott S. C. Wet Oxidation and Catalytic Wet Oxidation. Ind. Eng. Chem. Res. 2006, 45 (4), 1221–1258. 10.1021/ie051059n.

[ref21] Koh B.-B.; Lee E.-J.; Ramachandraiah K.; Hong G.-P. Characterization of Bovine Serum Albumin Hydrolysates Prepared by Subcritical Water Processing. Food Chem. 2019, 278, 203–207. 10.1016/j.foodchem.2018.11.069. 30583363

[ref22] Vaneeckhaute C.; Lebuf V.; Michels E.; Belia E.; Vanrolleghem P. A.; Tack F. M. G.; Meers E. Nutrient Recovery from Digestate: Systematic Technology Review and Product Classification. Waste Biomass Valorization 2017, 8 (1), 21–40. 10.1007/s12649-016-9642-x.

[ref23] Lu B.; Kiani D.; Taifan W.; Barauskas D.; Honer K.; Zhang L.; Baltrusaitis J. Spatially Resolved Product Speciation during Struvite Synthesis from Magnesite (MgCO3) Particles in Ammonium (NH4+) and Phosphate (PO43-) Aqueous Solutions. J. Phys. Chem. C 2019, 123 (14), 8908–8922. 10.1021/acs.jpcc.8b12252.

[ref24] Lin H.; Chen Y.; Shen N.; Deng Y.; Yan W.; Ruhyadi R.; Wang G. Effects of Individual Volatile Fatty Acids (VFAs) on Phosphorus Recovery by Magnesium Ammonium Phosphate. Environ. Pollut. 2020, 261, 114212 10.1016/j.envpol.2020.114212. 32109823

[ref25] Ion Exchange in Environmental Processes, 1st ed.; John Wiley & Sons, Ltd., 2017; 10.1002/9781119421252.

[ref26] Jorgensen T. C.; Weatherley L. R. Ammonia Removal from Wastewater by Ion Exchange in the Presence of Organic Contaminants. Water Res. 2003, 37 (8), 1723–1728. 10.1016/S0043-1354(02)00571-7. 12697216

[ref27] Huq N. A.; Hafenstine G. R.; Huo X.; Nguyen H.; Tifft S. M.; Conklin D. R.; Stück D.; Stunkel J.; Yang Z.; Heyne J. S.; Wiatrowski M. R.; Zhang Y.; Tao L.; Zhu J.; McEnally C. S.; Christensen E. D.; Hays C.; Van Allsburg K. M.; Unocic K. A.; Meyer H. M.; Abdullah Z.; Vardon D. R. Toward Net-Zero Sustainable Aviation Fuel with Wet Waste-Derived Volatile Fatty Acids. Proc. Natl. Acad. Sci. U. S. A. 2021, 118 (13), e2023008118 10.1073/pnas.2023008118. 33723013 PMC8020759

[ref28] Dong T.; Van Wychen S.; Nagle N.; Pienkos P. T.; Laurens L. M. L. Impact of Biochemical Composition on Susceptibility of Algal Biomass to Acid-Catalyzed Pretreatment for Sugar and Lipid Recovery. Algal Res. 2016, 18, 69–77. 10.1016/j.algal.2016.06.004.

[ref29] Laurens L. M. L. Summative Mass Analysis of Algal Biomass—Integration of Analytical Procedures: Laboratory Analytical Procedure (LAP); Report No. NREL/TP-5100-60943; NREL, 2016; 1118072, 10.2172/1118072.

[ref30] Van Wychen S.; Laurens L. M. L. Determination of Total Solids and Ash in Algal Biomass: Laboratory Analytical Procedure (LAP); Report No. NREL/TP-5100-60956; NREL, 2016; 1118077, 10.2172/1118077.

[ref31] Van Wychen S.; Ramirez K.; Laurens L. M. L. Determination of Total Lipids as Fatty Acid Methyl Esters (FAME) by in Situ Transesterification: Laboratory Analytical Procedure (LAP); Report No. NREL/TP-5100-60958; NREL, 2016; 1118085, 10.2172/1118085.

[ref32] Van Wychen S.; Laurens L. M. L. Determination of Total Carbohydrates in Algal Biomass: Laboratory Analytical Procedure (LAP); Report No. NREL/TP-5100-60957; NREL, 2016; 1118073, 10.2172/1118073.

[ref33] Villas-Bôas S. G.; Delicado D. G.; Åkesson M.; Nielsen J. Simultaneous Analysis of Amino and Nonamino Organic Acids as Methyl Chloroformate Derivatives Using Gas Chromatography-Mass Spectrometry. Anal. Biochem. 2003, 322 (1), 134–138. 10.1016/j.ab.2003.07.018. 14705791

[ref34] Kaspar H.; Dettmer K.; Gronwald W.; Oefner P. J. Automated GC-MS Analysis of Free Amino Acids in Biological Fluids. J. Chromatogr. B 2008, 870 (2), 222–232. 10.1016/j.jchromb.2008.06.018. 18603486

[ref35] Sato N.; Quitain A. T.; Kang K.; Daimon H.; Fujie K. Reaction Kinetics of Amino Acid Decomposition in High-Temperature and High-Pressure Water. Ind. Eng. Chem. Res. 2004, 43 (13), 3217–3222. 10.1021/ie020733n.

[ref36] Shende R. V.; Levec J. Wet Oxidation Kinetics of Refractory Low Molecular Mass Carboxylic Acids. Ind. Eng. Chem. Res. 1999, 38 (10), 3830–3837. 10.1021/ie9902028.

[ref37] Shende R. V.; Levec J. Subcritical Aqueous-Phase Oxidation Kinetics of Acrylic, Maleic, Fumaric, and Muconic Acids. Ind. Eng. Chem. Res. 2000, 39 (1), 40–47. 10.1021/ie990385y.

[ref38] Yousefifar A.; Baroutian S.; Farid M. M.; Gapes D. J.; Young B. R. Fundamental Mechanisms and Reactions in Non-Catalytic Subcritical Hydrothermal Processes: A Review. Water Res. 2017, 123, 607–622. 10.1016/j.watres.2017.06.069. 28709105

[ref39] Ding Y.; Sartaj M. Optimization of Ammonia Removal by Ion-Exchange Resin Using Response Surface Methodology. Int. J. Environ. Sci. Technol. 2016, 13 (4), 985–994. 10.1007/s13762-016-0939-x.

[ref40] Drissi R.; Mouats C. REMOVAL OF PHOSPHATE BY ION EXCHANGE RESIN: KINETIC AND THERMODYNAMIC STUDY. Rasayan J. Chem. 2018, 11 (3), 1126–1132. 10.31788/RJC.2018.1132081.

[ref41] Tarpeh W. A.; Udert K. M.; Nelson K. L. Comparing Ion Exchange Adsorbents for Nitrogen Recovery from Source-Separated Urine. Environ. Sci. Technol. 2017, 51 (4), 2373–2381. 10.1021/acs.est.6b05816. 28098981

[ref42] López-Garzón C. S.; Straathof A. J. J. Recovery of Carboxylic Acids Produced by Fermentation. Biotechnol. Adv. 2014, 32 (5), 873–904. 10.1016/j.biotechadv.2014.04.002. 24751382

[ref43] Johir M. A. H.; George J.; Vigneswaran S.; Kandasamy J.; Grasmick A. Removal and Recovery of Nutrients by Ion Exchange from High Rate Membrane Bio-Reactor (MBR) Effluent. Desalination 2011, 275 (1), 197–202. 10.1016/j.desal.2011.02.054.

[ref44] Jorgensen T. C.; Weatherley L. R. Continuous Removal of Ammonium Ion by Ion Exchange in the Presence of Organic Compounds in Packed Columns. J. Chem. Technol. Biotechnol. 2006, 81 (7), 1151–1158. 10.1002/jctb.1481.

[ref45] Williams A. T.; Zitomer D. H.; Mayer B. K. Ion Exchange-Precipitation for Nutrient Recovery from Dilute Wastewater. Environ. Sci. Water Res. Technol. 2015, 1 (6), 832–838. 10.1039/C5EW00142K.

