# Supplementary material for: Nutrient Recovery from Algae Using Mild Oxidative Treatment and Ion Exchange
Source: ACS Sustain Chem Eng. 2024 May 20;12(22):8573–80. doi: 10.1021/acssuschemeng.4c02658 (PMC11151276; doi:10.1021/acssuschemeng.4c02658)
Supplement: Supplementary file 1 — sc4c02658_si_001.pdf [file sc4c02658_si_001.pdf]

Supporting Information for:

Nutrient Recovery from Algae using Mild Oxidative Treatment and Ion Exchange

Tobias C. Hull [1,2], Kameron J. King[3], Jacob S. Kruger[2], Earl D. Christensen[4], Ali Chamas[2], Philip T. Pienkos[5], and Tao Dong[4*]

1. Advanced Energy Systems Graduate Program, Colorado School of Mines, Golden, CO 80401
2. Renewable Resources and Enabling Sciences Center, National Renewable Energy Laboratory, Golden, CO 80401 USA
3. Department of Civil and Environmental Engineering, Old Dominion University, Norfolk, VA 23529
4. Catalytic Carbon Transformation & Scale-Up Center, National Renewable Energy Laboratory, 15013 Denver West Parkway, Golden, CO 80401
5. Matereal, Inc. 1007 Savannah Ave., Pittsburgh, PA 15227

7 Pages

11 Tables

*Corresponding author: Tao.Dong@nrel.gov

Table S1. Amino acid profile of extracted algae solids. Amino acid profile was determined after acid hydrolysis by ion exchange chromatography with post-column ninhydrin derivatization. This analysis does not quantify methionine, cysteine, or tryptophan.

Amino Acid	mg AA / g biomass
Alanine	18.0
Arginine	12.7
ASX	9.1
GLX	27.1
Glycine	12.6
Histidine	4.6
Isoleucine	14.8
Leucine	28.0
Lysine	12.9
Phenylalanine	17.5
Proline	10.5
Serine	9.3
Threonine	11.8
Tyrosine	11.7
Valine	17.9

Table S2. Lipid profile of extracted algae solids measured as fatty acid methyl esters.

FAME	mg / g biomass
C8:0	0.20
C10:0	0.19
C12:0	0.17
C14:0	1.98
C15:0	0.09
C16:0	6.98
C16:1n11	0.43
C16:1n7	10.85
C16:1n6	0.00
C16:1n5	0.02
C16 other	0.61
C16:2	0.11
C17:0	0.05
C16:3	0.30
C16:4	0.31
C18:0	0.11
C18:1n9	2.49
C18:1n7	0.24
C18:2n6	1.54
C18:3n6	0.08
C20:3n6	0.18
C20:4n6	1.46
C20:5n3	7.85
C22:2	0.11

Table S3. Carbohydrate profile of extracted algae solids measured by HPAEC-PAD system with PA-1 column/guard. <loq indicates that monomer was present but below detection limits. N/D indicates that monomer was not detected.

Monomer	mg/g biomass
Mannitol	<loq
Fucose	<loq
Rhamnose	<loq
Galactosamine	<loq
Arabinose	<loq
Glucosamine	<loq
Galactose	3.1
Glucose	12.1
Mannose	<loq
Xylose	N/D
Ribose	<loq

Table S4. Numeric data for Figure 1. Reaction conditions: 0.5 g dry solids, 25 mL H_2O, 200 °C, 2 bar O2 partial pressure added upon reaching reaction temperature.

	C-Yield (% C)						N yield (% N)		
Time (min)	Formic acid	Acetic acid	Propionic acid	Succinic acid	Alanine	SUM C	Ammonium	Alanine	SUM-N
0	0.0	0.0	0.0	0.0	3.8	3.8	10.4	9.4	19.8
10	0.9	0.2	0.3	0.0	0.2	1.7	39.5	0.6	40.1
20	2.0	1.3	0.7	0.6	0.0	4.6	50.2	0.0	50.2
40	3.0	2.9	1.6	2.4	0.0	9.9	81.3	0.0	81.3
60	2.9	2.4	0.8	2.1	0.0	8.2	80.4	0.0	80.4
120	2.7	2.6	0.7	0.7	0.0	6.7	80.7	0.0	80.7

Table S5. Numeric data for Figure 1, expressed as product concentration rather than as carbon and nitrogen molar yields. Reaction conditions: 0.5 g dry solids, 25 mL H_2O, 200 °C, 2 bar O2 partial pressure added upon reaching reaction temperature.

	Product (mg/L)					
Time (min)	Formic acid	Acetic acid	Propionic acid	Succinic acid	Ammonium	Alanine
0	0	0	0	0	160	690
10	260	40	50	0	590	40
20	570	230	110	100	750	0
40	850	550	240	380	1210	0
60	820	460	130	320	1200	0
120	760	500	100	115	1210	0

Table S6. Numeric data for Figure 2a and 2b. Reaction conditions: 0.5 g dry solids, 25 mL H_2O 2 bar O2 partial pressure added upon reaching reaction temperature, 40 minutes reaction time.

	C-Yield (% C)						N yield (% N)		
Temperature [C]	Formic acid	Acetic acid	Propionic acid	Succinic acid	Alanine	SUM C	Ammonium	Alanine	SUM-N
175	2.1	1.0	0.5	0.6	0.2	4.4	25.5	0.5	26.0
200	3.0	2.9	1.6	2.4	0.0	9.9	81.0	0.0	81.0
225	1.6	2.2	1.1	1.7	0.0	6.6	82.4	0.0	82.4
250	0.9	2.8	0.9	1.9	0.0	6.5	81.0	0.0	81.0

Table S7. Numeric data for Figure 2a and 2b, expressed as product concentration rather than as carbon and nitrogen molar yields. Reaction conditions: 0.5 g dry solids, 25 mL H_2O 2 bar O2 partial pressure added upon reaching reaction temperature, 40 minutes reaction time.

	Product (mg/L)					
Temperature [C]	Formic acid	Acetic acid	Propionic acid	Succinic acid	Ammonium	Alanine
175	600	180	80	100	380	40
200	850	550	240	380	1210	0
225	450	410	170	270	1230	0
250	260	520	140	290	1210	0

Table S8. Numeric data for Figure 2c and 2d. Reaction conditions: 0.5 g dry solids, 25 mL H_2O, 200 °C, 40 minutes reaction time.

O_2 Pressure (Bar)	C-Yield (% C)						N yield (% N)		
	Formic acid	Acetic acid	Propionic acid	Succinic acid	Alanine	SUM C	Ammonium	Alanine	SUM-N
1	1.3	0.4	0.5	0.0	0.0	2.2	42.2	0.0	42.2
2	3.0	2.9	1.6	2.4	0.0	9.9	81.0	0.0	81.0
4	2.9	3.1	0.8	2.4	0.0	9.2	80.4	0.0	80.4
8	2.4	4.1	0.9	2.0	0.0	9.4	79.7	0.0	79.7

Table S9. Numeric data for Figure 2c and 2d, expressed as product concentration rather than as carbon and nitrogen molar yields. Reaction conditions: 0.5 g dry solids, 25 mL H_2O, 200 °C, 40 minutes reaction time.

O_2 Pressure (bar)	Product (mg/L)					
	Formic acid	Acetic acid	Propionic acid	Succinic acid	Ammonium	Alanine
1	360	80	80	0	630	0
2	850	550	240	380	1210	0
4	830	580	120	380	1200	0
8	700	760	140	310	1190	0

Table S10. Numeric data for Figure 2e and 2f. Reaction conditions: 2 bar O_2 partial pressure added upon reaching reaction temperature, 200 °C, 40 minutes reaction time.

	C-Yield (% C)						N yield (% N)		
Solids [g/L]	Formic acid	Acetic acid	Propionic acid	Succinic acid	Alanine	SUM C	Ammonium	Alanine	SUM-N
20	3.0	2.9	1.6	2.4	0.0	9.9	81.0	0.0	81.0
50	1.0	0.9	1.0	1.7	0.0	4.7	61.1	0.0	61.1
100	0.5	0.6	0.4	0.8	0.9	3.2	44.5	2.3	46.8
150	0.3	0.3	0.2	0.5	1.8	3.2	36.2	4.4	40.6
200	0.2	0.3	0.3	0.4	1.6	2.8	38.2	4.0	42.2

Table S11. Numeric data for Figure 2e and 2f, expressed as product concentration rather than as carbon and nitrogen molar yields. Reaction conditions: 2 bar O_2 partial pressure added upon reaching reaction temperature, 200 °C, 40 minutes reaction time.

	Product (mg/L)					
Solids [g/L]	Formic acid	Acetic acid	Propionic acid	Succinic acid	Ammonium	Alanine
20	850	550	240	380	1210	0
50	710	420	400	680	2280	0
100	780	570	320	590	3320	850
150	730	440	280	600	4050	2460
200	710	520	450	580	5700	2940
